# Catalysis as a Chemical, Physiological, and Pathological Process

**Published:** 1864-03

**Authors:** John Reid

**Affiliations:** Chicago, Ill.


ARTICLE X.

### CATALYSIS AS A CHEMICAL, PHYSIOLOGICAL, AND PATHOLOGICAL PROCESS.

BY JOHN REID, M.D., of Chicago, Ill.

Communicated to the Chicago Medical Society.

Catalysis is a term used to designate the changes produced among the proximate and elementary principles of a compound, by virtue of the presence, merely, of a substance which does not of itself enter into combination with them. In ordinary chemical reactions the substances brought together undergo mutual decomposition, and in the new combinations which take place all traces of the original compounds disappear. On the contrary, in catalytic changes, the active agent of the process contributes nothing of its own composition or elements to the substances acted upon; its mere presence being sufficient to induce them. No explanation can, in the present state of our knowledge, be given of the nature of the force thus brought into exercise, and hence, perhaps, the propriety of the designation employed, which simply expresses the action of "presence" in inducing decomposition.

The essential conditions of its action are, first, the presence of a compound substance capable of undergoing change, and second, the contact of a substance capable of exciting or setting those changes in motion. It is not necessary to the idea of catalysis that the catalytic agent should itself remain unchanged. This may or may not occur. Thus, where an inorganic compound, as sulphuric acid, is the catalytic, no alteration takes place in it. On the other hand, when it is an organic principle, in most cases, perhaps in all, changes originating in it, constitute the first and essential part of the process.

The class of phenomena comprehended under this term though obscure, is yet an extensive one, and of common occurrence. It is not limited to any particular set or group of substances. It may be seen in the inorganic kingdom in the action of one inorganic body upon another. Also in the action of an inorganic on an organic substance. It is most abundantly and familiarly developed in the action of azotised substances on each other, and on other organic substances, as in ordinary fermentation and other analogous changes. It also comprehends a large and most important class of vital phenomena, as the digestion of food, and, probably, its ultimate assimilation in the tissues, and the varied metamorphoses occurring there. In various pathological states we witness its direful operation, even in the mildest phases of its action, in contamination and perversion of the animal fluids, in disturbance of all the functions of life, and in its graver operation a suddenness of dissolution which no other morbid agency can rival.

It will be the object of this paper to show the operation of the catalytic force in the various departments mentioned, with a view to a clearer comprehension of its agency in the induction of what are called zomotic and catalytic diseases.

I will then first glance briefly at its operation among inorganic substances. It is well known to the chemist that oxygen and hydrogen may be kept mixed for any length of time at common temperatures without combination taking place. But on addition of platinum, either finely powdered or in the sheet, combination immediately commences, and water is formed. It is unnecessary to say that the platinum contributes nothing beyond its mere presence to this result. Other metals, as palladium and gold, and even glass, possess the same power, though in inferior degree. Second. If a little finely powdered peroxide of manganese is added to chlorate of potassa, and a gentle heat applied, oxygen will be set free at a far lower temperature than when chlorate of pot. alone is used. All the oxygen comes from the chlorate, which is completely decomposed, while an entirely different substance, the chloride of potassium, is formed. The peroxide of manganese remains entirely unchanged. Many other illustrations might be given of the operation of catalysis among inorganic compounds, but these will be sufficient. I will now proceed to show that inorganic substances not only induce catalysis in others of the same class, but that some of them have the remarkable property of inducing it in organic substances.

Thus, dilute sulph. acid with the aid of moderate heat, converts gelatinous starch into dextrine, a substance having different physical properties, but identical chemical composition. On the application of greater heat, the dextrine is transformed into glucose or grape sugar.

Again, cane sugar in solution with sulph. acid, is rapidly changed into glucose, a substance of different though allied composition, and possessing physical and chemical characteristics of an entirely different kind. Six equivalents of oxygen, and six of hydrogen, have by decomposition of the water present, been added to the composition of the original substance introduced. In both these experiments, the sulph. acid remains of course unchanged and undiminished.

These transformations are precisely what take place in the digestion of starch and cane sugar in the human body, where the gastric fluids and intestinal juices take the place of the sulph. acid in the experiments with corresponding results.

The action of many of our most valued and potent remedial agents, as mercury, iodine, arsenic, and others, are obvious instances of catalysis. No other remedies produce more decided and positive effects than these, yet they undergo no decomposition in the process, and are cast out unchanged.

The beneficent influence exerted by these agents in many forms of disease, and their equally destructive powers in cases unsuited for their administration, affords a wide and familiar field of observation, and present some of the most striking instances of the catalytic power of inorganic over organic substances.

Facts of this kind have many interesting physiological and pathological bearings, and can hardly with propriety be excluded from our consideration when we remember how largely and intimately inorganic substances enter into the composition of the human body, and the extent to which they may be introduced in the various ingesta and as medicines. Sulphur, we know, forms an essential part in the composition of all the protean substances, being incapable of complete separation from them, without destruction of the organic substance, and most of the various azotised principles which make up the bulk of the human body, are characterized by the presence of various inorganic substances which are essential to their composition. That their presence is essential is proved by their constancy, as well as by the intimacy of their combination, and it cannot be doubted that they take an important part in the metamorphosis of tissue. Whether the influence they exert is by that of ordinary chemical attraction, or that of catalytic power, cannot as yet be positively stated, but that it is by the latter is not an im probable assumption, in view of the facts which I have stated, and many others which might be presented.

It is, however, among organic substances that the operation of catalytic force is more particularly and familiarly seen. One class of these, the non-azotised, though permanent under ordinary circumstances, are characterized by a special aptitude to undergo decomposition in the presence of a catalytic or ferment. The other, the azotised, are equally characterized by the property they all enjoy in an extraordinary degree, when in process of decay, of exciting these changes in proximate principles and in each other. Hence their designation in reference to these changes of catalytics or ferments. The most familiar illustration of these facts is seen in the process of fermentation. A solution of sugar in water has no tendency to ferment, but on introducing an azotised body, as gluten, the process is immediately set in motion, resulting, as we all know, in the transformation of sugar into alcohol and carbonic acid, the gluten undergoing change, but contributing nothing of its elements to this result.

The substance yeast, usually employed in the fermentation of beer, is dependent for its powers on the gluten it contains. Examined under the microscope, it is found to abound in minute transparent vesicles containing granules, which are believed to be living infusory plants, which have the power, and do actually propagate themselves, at expense of proximate principles in the solution, in which they induce fermentation. We have thus two processes going on side by side in the solution, a change in the ferment which impresses similar changes upon all proximate principles present, assimilating them to its own nature, and decomposition of the sugar, out of the elements of which alcohol and carb. acid are formed.

Fermentation, then, as we have seen, is but a catalytic process. The same conditions are required for its developement, and the results are of the same kind.

The visible extrication of carbonic acid gas occurs in vinous fermentation; but this extrication is not essential to the idea even of fermentation. In the case of acetous fermentation, although as often conducted, carbonic acid is given off, it is not essential, (fixation of oxygen from the air being the essential change,) and in the improved processes of forming vinegar from alcohol it does not take place.

The conversion of glucose and milk sugar into lactic acid under the action of a ferment, is as true an instance of fermentation as the conversion of cane sugar into alcohol and carb. acid. The state of intestine motion and change which the term fermentation requires is met in the decomposition altered relation of atoms and change of structure which takes place.

The exception, then, which has been taken to zymosis, as a term used to express certain morbid processes which take place in the human body under the operation of a morbid poison, on the basis that fermentation, which the word Zymos is meant to express, requires the evolution of carb. acid, is therefore groundless. Webster defines fermentation as follows:—In its most general sense it may be defined any spontaneous change which takes place in mineral and vegetable substances after life has ceased. And a later authority, Chalmers' Encyclopediæ, says: "Fermentation is a term applied to the change which occurs in the organic substance, when influenced by another in a state of decay." For those who prefer it, the word catalysis may be substituted for zymosis with propriety, although the phenomena of increase of the catalytic agent or morbid poison which takes place in some truly contagious and infectious diseases, as small pox, measles, &c., renders zymosis the preferable term.

Before leaving this part of the subject, we might well challenge those who take exception to the word zymosis on the basis mentioned to show that no carbonic acid is actually evolved in the diseased changes in question. We know this to be the most constant and abundant product of tissue metamorphosis in a state of health, and therefore may, by the fairest deduction, be considered to take place in the quickened metamorphosis of catalytic decomposition.

I have thus diverged from my main subject to answer some objections which stood in the way of a profitable discussion of zymotic diseases at our last meeting. I will now return to the subject of catalysis as acting among organic substances. It will probably be unnecessary to illustrate the subject farther than has been done under the head of fermentation. I will now consider it as a part of vital phenomena in various physiological processes. An azotised substance called diastase is easily obtained from malt by maceration in lukewarm water, which has remarkable catalytic power over starch, converting it with great rapidity into dextrine and glucose; thus producing the same effects as we have seen result from the presence of sulphuric acid. This substance, diastase, is always found in small quantity in germinating seeds and buds, in the act of development. As the process of germination advances, the starch of the seed disappears, and dextrine and glucose are formed, as is manifest without chemical tests, in the sweet taste of the juices of the young plant, and their gummy feel. Is there not good reason to believe that in this physiological act, the diastase is the active agent of the process, and that it produces these important changes in the constituents of the seed in the same way that it does upon these same constituents elsewhere.

In the saliva of man there is present an azotised substance called ptyaline, upon which the peculiar digestive power of the saliva upon starchy food depends. So energetic is its action that if starch paste, at a temperature of 100° F., is introduced into the mouth, it will yield traces of sugar in half a minute. The admixture of starch and saliva out of the body produces the same effect, and is a true instance of catalysis. But it is in the small intestine that the digestion of starch and sugar especially takes place. Under the action of the intestinal juices, the same series of changes take place which we have seen under the catalytic action of sulph. acid and diastase. The starch is converted into dextrine, the dextrine into glucose, in which state it passes into the circulation. The active agents in inducing these changes are found by experiments both in and out of the body to be the pancreatinim, a peculiar azotised principle of the pancreatic juice, and the peculiar secretion (not yet well analyzed) of the follicles of Brunner and Lieberkuhn: these substances yielding nothing of their own elements to the new substances formed, evidently act by virtue of their catalytic power.

The process of stomach digestion gives us another remarkable evidence of catalytic transformation. Under the action of the gastric juice, all the albuminoid elements of the food, as albumen, fibrin, musculine, caseine, &c., are without distinction converted into a new substance called albuminose. This substance, while it has the general characters of those of the azotised class in being uncrystalizable and containing nitrogen, has peculiar characters of its own. Unlike albumen, it is not precipitated by nitric acid and heat; and has the property of freely permeating organic membranes, which albumen has not. The process of stomach digestion, then, is not a simple solution of albuminoid substances, but a transformation of them, effected by the gastric juice. This power of transformation does not reside in the acids of the gastric juice, which are found at the temperature of the human body to exercise only a feeble influence, although at the point of ebullition they possess it. The active agent in the process is found in the pepsine, a peculiar azotised substance found in the stomach, which acts the part, even in minute quantities, of a most energetic ferment. So energetic is its action that half a grain of acetate of pepsin dissolved in hydrochloric acid is capable of taking up 210 grains of white of egg, while the acid alone, at the same temperature, had an inappreciable effect. The azotised food being thus reduced to the condition of albuminose, the digestive process is perfected, and in this form it freely permeates the membranes and enters the circulation.

We have thus seen that the various alimentary substances, both of an azotised and non-azotised kind, required for the sustenance of the body, are fitted for appropriation by a process of catalysis. That in each kind of digestion there is provided a special catalytic agent, with special powers over the particular substances subjected to its agency. These and other considerations would naturally lead us to the inference that in the progress of these alimentary substances, thus changed, towards the general circulation, the changes which take place in them, and their subsequent metamorphosis in the blood, are the results of a similar process. Here we are no longer able, as in the former ease, to give a positive demonstration of the nature of the changes which occur. We are left more to inferences and deductions, and the convincing power of analogies. These, however, establish, on a tolerably firm basis, that the same force is in exercise in the latter case as in the first. And further, that the whole process of final assimilation in the tissues, and the various metamorphoses occurring there, both of a progressive and retrogressive kind, are referrable to its operation.

It would obviously be impossible in a brief article to enter into anything like a full description of these subjects, even if our knowledge of them warranted it. I will only therefore allude to a few of the least debatable instances of catalytic transformation occurring in the penetralia of the body.

In examining the blood of the portal veins we find its albuminous constituent to exist in the form of albuminose. After emerging from the liver, it is found in the hepatic veins and vena porta ascendeus, no longer as albuminose, but as albumen. This change, according to Carpenter, has been effected by contact with the tissues of the liver. The catalytic agency of that organ, or some unascertained principle in it in inducing this change, seems evident. A similar catalytic influence is exerted by that organ in the glucose which it receives from the portal vein after the digestion of starchy food. The saccharine principle, entering as glucose, emerges in the hepatic veins as liver sugar, a form of sugar of which the blood is much more tolerant than of any other. Again, the liver is proved to be of itself a sugar-producing organ. It is capable of producing it from albuminoid substances. Of the various decompositions and metamorphoses which culminate in the production of liver sugar from albuminous substances we know nothing, but in the process of its elaboration we do know that a peculiar substance is formed called glycogene, intermediate in composition between dextrine and glucose—the formation of which is one step antecedent to the production of sugar. It does not of itself contain sugar, but is readily converted into it by contact with any of the animal ferments, as those contained in the saliva, or in the blood. The unavoidable inference in reference to the glucose conveyed to the liver, and converted there into liver sugar, is that it is so converted by a catalytic change similar to that by which cane sugar is converted into glucose by the action of a ferment; and in reference to the glycogene, that it is transformed into sugar in the same manner that dextrine could be transformed under similar conditions.

Both glucose and liver sugar rapidly disappear as such when introduced into the general circulation. LEHMAN and ROBIN consider that it undergoes immediate conversion into lactic acid, a substance of similar composition, and of the existence of which as a normal ingredient of the blood there is now no doubt, in which state these saccharine principles are appropriated to their peculiar purposes in the body. The facility with which these substances are transformed into lactic acid in the presence of azotised compounds out of the body, renders it extremely probable that a similar change is constantly going on under similar influences within it. The albuminous and fatty contents of the lacteals as they pass on to join the general circulation, under the catalytic agency of the vitalized surfaces over which they pass, are gradually prepared for their final destination, and after being subjected to the action of the air in the lungs, are finally mingled with the blood in the general circulation, where by the catalytic action of its ingredients, they are assimilated to its own composition.

Were we able, it would be interesting to follow these nutrient principles to their ultimate destination in the tissues, and mark the process of their transformation there into the vital constituents of the frame. But in these recesses we come into immediate contact with the principle of life itself. We cannot isolate the active agent, but the transformation is apparent, and so far as we see it is strictly catalytic. Each particular tissue or organ attracts to itself that which it requires for its nutrition, and once in contact with it transforms it into its own structure.

Then commences the destructive or retrograde metamorphosis, by which parts which have fulfilled their functions are prepared for expulsion from the body by transformation into effete and excrementatious products. These, under ordinary circumstances, are removed as fast as they are formed by the various excretory processes. If, from any cause, they should accumulate in the blood, serious derangement of the health, and even death, is sure to result. The noxious effects of retained carbonic acid and urea are sufficiently familiar. But independent of the directly poisonous influence which they exert, the retention of excrementitious products have been clearly demonstrated to establish a predisposition to the developement of various acute specific diseases. In proof of this I need only cite the prevalence and malignity of these disorders among persons breathing an "atmosphere charged with carbonic acid, and laden with putrescent emanations." "And the peculiar liability of the parturient female, whose blood is charged with substances in a state of retrograde metamorphosis, to that most fatal zymosis, puerperal fever." The liability of the intemperate, the famished, and the overworked to these diseases, may all be found in the contamination of the blood from the presence of excrementitious products. That these substances, already in a state of change, should be readily and powerfully acted upon by the introduction of an energetic ferment from without, is not at all surprising; or, that without any such introduction, some potent principle might be developed among its azotised constituents which might thus originate *de novo* some similar form of disease.

For the production of a certain class of contagious diseases, as the eruptive fevers, it seems to be well established that two conditions are essential. In the first place, an existing predisposition in the subject, which we call a predisposing cause, and in the second place, the inception of a morbid poison, which constitutes the excitor or exciting cause. If either of these are absent, the specific disease is not induced. These are precisely the conditions required for the production of catalytic action in general. A solution of sugar in water has no tendency to undergo decomposition until the catalytic agent or ferment is introduced. Again, in these contagious diseases, a surprising multiplication of the original morbid poison takes place during their progress. This also has its complete analogy, as we have already seen, in the development of the yeast plant, used to induce fermentation of an infusion of malt. The changes which actually occur in the blood in catalytic diseases furnish the last required analogy to the fermenting process. These are various and striking, but as yet have been but partially elucidated.

Its chemical analysis seems to be attended with peculiar difficulties, so that the least satisfactory of our knowledge of the pathology of disease, is that which concerns the blood.

In general it may be said that in typhoid, putrid, and malignant forms of disease, the fibrine is notably diminished, and also the other solid constituents. In the exanthemata and typhus, the alkaline salts are relatively increased. In the malignant forms, and in cholera, the coagulating power of the blood is much diminished, and sometimes destroyed.

These are not put forward as the peculiar or exclusive results of the operation of catalytic poisons; they are nevertheless results which attend their action. The arrested secretions and altered excretions afford more decided evidences of its perverted condition. The increase of urea, uric acid, hippuric, sulphuric, and phosphoric acids and the pigments, and the occasional presence of unusual products, as leucine and others, in the urine, indicate the presence of these effete matters in the blood in unusual proportions.

The presence of the coloring matters, sometimes in fourfold increase, is especially significant of destructive changes in the blood, being by the best investigators attributed to the disintegration of the blood corpuscles. The peculiar and characteristic emanations from the lungs and skin, and the strangely vitiated and often putrescent discharges from the bowels, all point directly to the blood out of which they are drawn or elaborated, as the primal source of their perversion. The poisonous quality of such blood introduced into the healthy organism, is better evidence of its perversion than any chemical examination could afford. A new and important sort of evidence of blood contamination in catalytic diseases has been furnished by the experiments of SCHMIDT. "He has discovered that blood drawn from the veins of a healthy man will not cause either sugar, urea, amygdalin nor asparagin to ferment. If the same blood be exposed to the air for a few days, it will occasion fermentation in saccharine matter, and after fourteen days' exposure another principle will be developed capable of causing that change both in urea and asparagin. On the other hand, blood drawn from persons laboring under various diseases of this class, including cholera, induces fermentation in a few hours, not only in sugar and urea, but also in amygdalin." These facts, brought prominently forward by Dr. POLLI, are conclusive as to the alterations in the quality of the blood, and complete the parallel sought to be established between the morbid processes at work in the diseases under consideration, and those of catalytic actions in general, and abundantly justify the designations of catalytic and zymotic, which have been applied to them.

